# Medical Report of the Internal Diseases Treated in the Royal Seraphim Hospital in Stockholm during the Year 1842

**Published:** 1846-10

**Authors:** 


### Art. XI.

*Redogörelse för Sjukvården å Kongl. Seraphimer Lazarettets Afdelning för Invertes Sjuke, under Loppet af Ar* 1842.

*Medical Report of the Internal Diseases treated in the Royal Seraphim Hospital in Stockholm, during the Year* 1842. By Dr. Magnus Huss. —*Stockholm,* 1844. 8vo, pp. 228.

We had imagined, on reading the title of this book, that it would be a mere hospital report, full of long details of cases, but with little attempt at generalization, and, consequently, merely valuable as an addition to medical statistics. From the charge—often well-founded—of deficient knowledge of contemporary medical literature and science, we knew our author would be perfectly free; but we were not prepared to find, under the modest name of an Annual Report, such elaborate disquisitions on the origin and nature of some of the most obscure diseases;—and these written, too, with a candour and honesty of purpose, which, in our own insular pride, we rarely expect to meet with beyond the British seas.

Before examining this Report in detail, we may premise that it is only a portion or single part of an elaborate series, several volumes of which are already before the Swedish public, though they are as yet unnoticed in our British Journals. Unfortunately these preceding volumes are not as yet in our possession, and this is the more to be regretted, as several maladies and classes of diseases which, to judge from the number of cases briefly alluded to in this volume, are of very frequent occurrence in the Swedish capital, obtain in this Report only the mere notice of their number, while, for details of their peculiarities, and for the author's opinions of their nature and treatment, we are referred to the preceding volumes.

In this Report of 1842, the various diseases that affect the human frame are considered successively under the following heads:

1. Diseases of the nervous system.
2. „ respiratory organs.
3. „ organs of circulation.
4. „ lymphatic system.
5. „ digestive organs.
6. „ liver and of the spleen.
7. „ urinary and genital organs.
8. „ organs of locomotion.
9. „ skin.
10. Mental disorders.
11. Cases of poisoning.

In the clinical wards of the Swedish hospital the most important diseases are necessarily selected for the instruction of the pupils, but those also of less intensity but of more frequent occurrence, are admitted, to familiarize the student with the aspect of maladies most common in his future practice. During the year 1842 seventeen students were admitted as clerks in the wards (tjenstgörande), and to each of these clerks were allotted, in the space of nine months, 35 cases of disease, of all of which they were obliged to keep a full and complete journal for the inspection of the clinical professor. Besides the clinical observations delivered by the professor at the bed side, a course of special pathology is continued throughout the session in lectures which are given twice in the week; and we can estimate the comprehensive nature of this course from the fact, that the nine months of the past session were exclusively occupied by the consideration of diseases of the intestinal canal, so that two or even three years must elapse, ere all the maladies of the human frame are completely described. The mortality in the hospital, during 1842, was $11\frac{1}{2}$ per cent.: the greatest number of patients died in the earlier months of that year; while December presented the lowest rate of deaths.

No epidemic of any consequence appeared in the capital during this year, excepting a gastric nervous fever among the police or gendarmerie, which increased the usual average of that complaint by about 60 cases.

The first division, viz. that of diseases of the nervous system, occupies a very large portion of this volume; but we do not regret to find more than one hundred pages devoted to these interesting but most obscure maladies. Our author commences with inflammation of the brain and spinal cord, and of their envelopes. The first case, which is related at great length, is one of fatal inflammation of the spinal meninges. The patient, a sailor, was able to perform his usual duties for fourteen days after the accident, before any serious symptoms supervened. No disease was discovered on dissection of the brain after death, but there was slight delirium during life, with great irritability of temper. Convulsions occurred, both in the upper and in the lower limbs, and it was necessary to use the catheter, as the bladder did not act. From the appearances recorded on the post-mortem examination, it would seem that the dura mater was the original seat of the inflammation, and that pus had exuded from thence into the neighbouring muscles on the back; though, during life, the latter phenomenon could not be distinguished from a simple abscess, the result of supposed external injuries. Dr. Huss ascribes the delirium and irritability of temper in this patient, to sympathetic irritation of the cerebral system. Death ensued in the fourth week after the accident.

The second case is not in itself remarkable, being one of simple *meningitis cerebralis,* but it was accompanied with a general emphysema of the whole trunk, which came on on the twenty-second day of the disease: no cause for this appearance could be discovered on dissection. Dr. Huss aptly compares this emphysema to that which occurs in typhoid fever of a malignant character, where the intestines become enormously distended with flatus. The cause of this symptom may, he thinks, in both cases, be traced to diminished energy of the inflamed brain, whereby the blood becomes deteriorated, and tends to separate into its constituent parts. Our author, however, evidently does not mean to infer that, in all cases of typhoid fever with great distension of the bowels, there is necessarily inflammation of the brain or of its envelopes.

In the third case of this Report (p. 9) we have an illustration of the great advantages of full doses of calomel after venesection in acute cerebral meningitis. The treatment here pursued seems to have been most energetic and sensible; it is one of those cases, where no doubt can be raised, as to the powers of medical science in arresting disease.

It is justly remarked by Dr. Huss, in reference to this case, that we must not be deterred by the apparent debility or defective nervous energy, from pursuing a steady antiphlogistic course of treatment. "It is this horror of debility," says he, "which too often paralyses the efforts of the physician, and allows the inflammation to proceed unchecked to a fatal termination." The great value of free evacuation of the bowels in cerebral disease, appears to be better known to Dr. Huss than to many of his continental brethren. "Should calomel," he says, "fail of purging, I assist its operation by purgative enemata, and by the free use of croton oil, or of turpentine." Our author has seen little benefit from the cold douche applied to the head in cerebral inflammation, but he acknowledges its marked efficacy in the delirium of typhus fever. At the same time he admits that there is great difficulty in the diagnosis between the two affections, as the blood often presents the same disintegrated condition in cerebral inflammation, as in the most malignant typhus. Blisters to the shaven scalp he unequivocally condemns; in all cases in which they have been tried, he has found them to do harm by increasing rather than diminishing the symptoms; though in the later stages, when inflammation has subsided and effusion still remains, they may occasionally be useful. "When," he continues, "the symptoms of inflammation and congestion have subsided, and are succeeded by those indicating debility of the brain and of the nervous system, I find it best to wait a day or two, till there is an obvious indication for the use of stimulants and sedatives; I then employ camphor and arnica to fulfil the former intention; and opium seems to be best adapted for the latter. I have more than once observed, that opium in doses of one or two grains when muttering delirium succeeds to violent nervous excitement, has quickly produced refreshing sleep." English practitioners will fully appreciate the truth of this observation.

Two cases of *hydrocephalus internus*, the next disease here mentioned, are not of remarkable interest, except that the first was found to be accompanied by a large cavity in the cerebellum containing fluid, and totally distinct from that found in the ventricles. He compares this appearance to the sacculated dropsies of the ovary, and believes these cysts to be formed by a defect in development of the cells of the cerebral substance. Dr. Huss has never observed any connexion between disease of the cerebellum, and the excitement or depression of the sexual appetite.

*Apoplexy*. Of 17 cases, 12 occurred in males, and 5 in females; and nine of these patients were between their thirtieth and fortieth year, which is surely a greater proportion than that met with at the same period of life in this country. Of these 17 cases, 8 proved fatal; in the preceding year only 4 died out of 15.

Our author has seldom found blisters or irritant applications to the scalp of any use after apoplexy, and the possible benefit they may confer is, he thinks, more than counterbalanced by the annoyance they occasion to the patient.

Dr. Huss does not believe that, when the power of speech is entirely lost in apoplexy, the effusion must necessarily have taken place in the anterior lobes of the brain; he has seen speech wanting when the posterior lobes alone were the seat of the maladies. In case 8 the apoplectic effusion occurred in the posterior lobes of the left side, and the power of speech was entirely absent during the six months that the patient survived the attack; but we must observe that the left hypoglossal nerve was found to be only half the size of that upon the opposite side.

Our author is of opinion that, the larger the clot and effusion of blood in apoplexy, the greater will be the loss of motor power, but he acknowledges that the same does not hold good in regard to the seat of the lesion; but, he adds, it is well known that the case is different in apoplexy occurring at the base of the brain, where a very small effusion causes great loss of nervous power.

At page 30 of the Report we find an interesting case exceedingly well detailed, which, during life, was supposed to be apoplectic effusion into the pons varolii. There was paralysis of both sides of the trunk, with partial loss of feeling, but the intelligence remained perfect. After death an effusion of blood was found in the left crus cerebri, and another of like character in the corpus striatum of that side. Dr. Huss owns that he dares not venture on any explanation of the double paralysis in this case.

Before concluding this portion of our subject we may observe that our author throws out a suggestion, that an intimate connexion will, one day or other, be discovered between lesion of the corpora striata and optic thalami, and the functions of the urinary and genital organs.

Under the title of, *Organic lesions of the brain* (p. 33), we find a case of supposed softening of that organ, with paralysis of the right side, spontaneous movements of the whole of the extremities, and loss of feeling in the fingers and toes. Two grains of opium divided into four doses were given daily, with ten grains of the "sal cornu cervi," by which combination our author believes that the narcotic properties of the opium are diminished, leaving its sedative qualities untouched. Dr. Huss has found opium very useful in cases of suspected softening of the brain.

> "I have constantly observed," he says, "that when in this complaint the most prominent symptoms were cramps of the limbs, or tremors, or both of these symptoms together, opium was the most efficacious remedy, though I cannot venture to assert that it effected a cure." (p. 37.)

*Osteoma frontalis.* In speaking of this disease and of tumours in general, pressing upon the surface of the brain, Dr. Huss observes, that it is difficult to decide *after death* what portion of the cerebral system has particularly and secondarily suffered from such pressure, though it is easy to determine what convolutions have been immediately compressed. The case of frontal osteoma here described, closely resembles those cancerous tumours described by Walther and Cruveilhier, and so beautifully figured by the latter. The pulsations of the brain could be very distinctly heard on applying the stethoscope over the tumour. How far the congested and enlarged vessels in the tumour itself may have contributed to the sound of cerebral pulsation, is a question which we leave to the consideration of our readers. It may be doubted, perhaps, in the present instance, whether we are justified in identifying the tumour described by Dr. Huss with those of a decidedly cancerous character; for its external portion diminished, in a very marked degree, under the prolonged use of mercury and iodine; while, at the same time, the mental powers which were before much weakened, regained their original force. The mode of treatment was, by first blistering the tumour and then applying the unguent. hydrarg. iodid. The tumour did not entirely disappear, but Dr. Huss suspects, from the complete return of the mental faculties, that the internal portion of it, which produced all the evil symptoms, was that which was soonest and most completely removed.

Spinal Irritation. To the consideration of this disorder Dr. Huss devotes a very considerable portion of this volume. It is indeed a malady concerning which much has been written lately, but we have seldom or never met with a more complete history of its varied symptoms than that contained in the pages of this Report. Our author complains of the numerous, varied, and complex definitions attempted of this disease; his own, however, will probably admit of dispute. He believes, "that that same condition of irritation which, physiologically speaking and under physiological influences, is transplanted from impressions made on the surface-nerves of the body to the spinal cord, here appears as a disease arising primitively from the spinal cord itself, and which is thence propagated to the external surface, or extremities of the nerves." Physiological irritation, therefore, is transmitted from without to the nervous centres, pathological irritation from the centres to the extremities of the nerves.

Dr. Huss asks, if this be not an old disease under a new name? or at least he acknowledges, that were many of the symptoms to be taken separately they have undoubtedly been before described; but the honour of uniting them all as one malady is due only to the present day. The subject of spinal irritation has been most ably discussed by English writers; indeed we may justly assume to ourselves much of the light that has been recently thrown on this obscure disease. The labours of the Messrs. Griffin, of Limerick, in this regard, are pleasingly acknowledged by Dr. Huss, who seems also to be well acquainted with the investigations of Dr. Marshall Hall, of Dr. Copland, and of Mr. Travers, as to the connexion of reflex action with the phenomena of the disease in question. Nor have our German brethren been slow to pursue a subject so congenial to their laborious and speculative minds. Our author has, we think, steered a middle course, a thoroughly practical one, between the mazes of German speculation and the hasty conclusions too often arrived at by the clever writers of France. We do not think we say too much when we state his views and practice here will be found as consonant to English feelings and to English medicine, as is the construction of the beautiful language of Sweden to that of our mother country.

Dr. Huss objects to the generally received definition of spinal irritation, viz. that it is an alteration in the *functions* of the spinal cord, independent of any organic change in that part; for he observes, that this merely expresses the condition and not the original cause of the malady; pathological anatomy, he acknowledges, has taught us but little regarding its origin, and that little merely of a negative character, viz. that the same phenomena which are developed in organic disease of the spinal cord, may also appear during life, when no lesion or change of structure can be discovered on dissection.

The symptoms of spinal irritation are classed by our author under the following heads:

1. Pain of various parts of the vertebral column, existing either idiopathically or developed by pressure. 2. Cramps, either of a tonic or of a clonic nature, in those parts subjected to the influence of the spinal cord. 3. Loss of power in the same portions of the body, varying from simple stiffness and weakness to complete paralysis. 4. Altered sensibility, either by excess or by great diminution of sensation.

Of all these phenomena the varieties seem to be almost endless; and not less remarkable is the constant variation of symptoms in the same individual. We will, however, briefly consider each of these divisions as here made out more in detail.

1. Dr. Huss believes that the pain developed by pressure on the vertebræ is of more importance, in a diagnostic point of view, than that experienced by the patient without such manipulation; but he differs from some of our recent writers, when he asserts that it is only the feeling of pain developed by *firm* pressure that is of importance. With the Drs. Griffin he agrees that pain *above* the vertebræ, subjected to pressure, is rarely observed. It is almost always felt in those organs or parts of the frame that correspond in position to the compressed vertebræ; or, if not there, it occurs in more distant parts lying *below* the seat of pressure. But Dr. Huss does not maintain that tenderness on pressure of the vertebræ must necessarily exist in spinal irritation. On the contrary, it is occasionally absent; and its value as a diagnostic sign is considerably diminished by its occurring, with a similar train of symptoms, in organic disease of the spine itself. As to the immediate cause and nature of this symptom, Dr. Huss rejects alike the theory of its being occasioned by pressure on the spinal cord through the medium of the vertebræ, which is anatomically impossible, as also the doctrine of Stilling and of Hirsch, who believe the seat of the pain to be in the muscles, skin, and ligaments interposed between the vertebræ and the hand of the compressor. Our author finds no other means of explaining this symptom than by supposing that there exists in such cases a neuralgia of the vertebræ themselves. This theory may perhaps suffice in the present state of our knowledge to account for such intense pain being felt on firm pressure of the vertebræ; nor do we think it inadequate to explain those cases where the slightest touch occasions agonizing torture, and even convulsions. The theory of a neuralgic condition of the sensitive surface-nerves of the back must be also allowed to carry with it a considerable semblance of truth.

2. "Phenomena resembling cramp," &c. From the simplest attacks of cramp these symptoms may pass into severe convulsive movements of the extremities, closely resembling those of chorea; which disease our author considers to be spinal irritation, in its most complete condition, with clonic convulsive movements. At other times the symptoms are rather those of tetanus; and this latter malady is, according to Dr. Huss, the type of spinal irritation in its tonic and most acute form.

3. "Diminished power of motion." General paralysis of the motor powers sometimes occurs under this form.

4. "Altered sensibility," &c. This may be diminished, or much increased. In exalted sensibility, accompanying spinal irritation, the cutaneous nerves are often so affected in very limited portions of the surface; sometimes this does not exceed the space covered by a shilling. Dr. Huss looks upon spinal neuralgia as another variety of this form. On the other hand, sensation may be totally absent from some portions of the cutaneous surface, even to impressions of the most painful nature.

Of the difficulties attending the diagnosis of spinal irritation our author speaks most feelingly; and he acknowledges that in many cases of obscure disease affecting the spinal column, it is necessary to suspend your opinion for weeks, nay, sometimes even for months, to enable you to decide on the nature of the malady, from the effects, or from the negative results, of the remedies employed.

Dr. Huss adds but little to our previous information respecting the causes of this malady, but ascribes its prevalence among the females of the higher classes in Sweden to that false system of female education, which neglects the due exercising of the body, while it overstrains the mental powers. Whatever, then, diminishes the bodily strength, as overstudy, excitement, or long and debilitating disorders, will predispose to this disease; especially in nervous and irritable constitutions. Of immediate exciting causes our author reckons the following: (*a*) Contusions, strains of the spinal column, from lifting of heavy weights; (*b*) intense mental affections, fear, sorrow, &c.; (*c*) lesions and irritations of the extremities of nerves springing from the spinal column, both of those distributed on the cutaneous surface, and those which terminate in internal organs, as also the presence of worms in the intestinal canal; (*d*) the most important and most frequent cause of spinal irritation is a disordered state of the blood itself (giving rise to the neuræmia of Dr. Laycock). As an example of this, Dr. Huss refers to the frequent occurrence of spinal irritation in chlorotic females, and to the phenomena produced by poisoning with strychnia, or secale cornutum, or by the introduction of lead into the system.

As regards the termination of this malady, it may persist for years without any advance towards a cure, the only change being the constant variation of the symptoms; but it may also eventually produce organic disease. Thus constant palpitation of the heart, from spinal irritation, may eventually cause the dilatation of that organ, or more frequently hypertrophy, with or without dilatation. With our English writers, Dr. Huss agrees that spinal irritation of itself is rarely fatal.

A long paragraph is devoted by our author to a review of the theories of spinal irritation. All that have hitherto been broached he rejects as doubtful and unsatisfactory, but modestly enough he does not attempt to replace them by a theory of his own. Perhaps he inclines a little towards the theory of Hirsch, whose opinions in regard to the minute anatomy of the nervous system are somewhat analogous to those of Dr. Stark, of Edinburgh. Hirsch believes the nerves to be tubes containing a fluid which, when collected in excess in any part of the nervous system, gives rise to pain, and to the other symptoms of spinal irritation; thus assimilating these phenomena closely to congestion of the blood-vessels. But, as Dr. Huss very justly adds, "until the existence of this nerve-fluid, or ether, or essence, can be distinctly proved, these doctrines may amuse but cannot instruct the scientific pathologist."

*Treatment.* External remedies should be applied in general to that part of the spine where pain is felt on pressure. Venesection, whether local or general, should be cautiously employed in debilitated persons, where the disease, as in chlorosis, appears to be connected with deterioration of the blood; and Dr. Huss remarks that venesection alone is seldom able to effect a cure, but at the most acts only as a palliative of the disease. Of the class of derivatives, Dr. Huss employs most of those generally used in English practice, recommending always that we should begin with the milder forms, while in protracted cases he has employed moxas and the actual cautery with success. "Should the patient seem worse after using the milder derivatives, I always abstain from having recourse to those of a severer kind."

Much alleviation of the pain is often obtained by simple manipulation or shampooing of the affected parts; and our author has also seen great benefit to result from covering the spine with wool or cotton dipped in turpentine. This is kept applied to the back by means of a sheet or towel, and its moisture is preserved by continual irrigation with fresh turpentine.

This plan is, however, not without danger in highly irritable constitutions. Mercurial ointment, pushed to salivation, has proved occasionally useful in our author's hands, though no inflammatory symptoms were present. Though no disciple of Preissnitz, Dr. Huss states that he has seen favorable results from the cold douche, and from cloths steeped in cold water, and applied to the spine; but he acknowledges that in the majority of instances these remedies have proved injurious.

Electricity and electro-galvanism are contraindicated, he thinks, in all cases where plethora exists, but may be employed with advantage in chronic and debilitated cases, though the advantage resulting from their use is so slowly obtained, as to render their application most tedious to the patient. In cases of a neuralgic character, magneto-electricity combined with acupuncture caused a most marked improvement in the symptoms.

1. *General external remedies.* "Bathing," &c. The warm bath is occasionally useful, but is much more efficacious when ℥j of caustic potash is diffused in the water. Perhaps, however, as our author suggests, a smaller quantity of the alkali than that above stated should be first tried, and the baths should be repeated at first every third, and afterwards every second, day. The mud-baths of Germany (soolenbäder) are often really valuable in cases of spinal irritation; and Dr. Huss deplores the little attention paid in general to the baths and mineral springs of his own country, which, he believes, would be found to be as efficacious as many of the celebrated German spas, if his countrymen would but be convinced that the means of cure were to be found at their own doors.

*Clothing.* Much harm is done, according to our author, by the habit of wearing excessive quantities of warm clothing. Thus he relates an instance of a lady, who suffered from spinal irritation, with constant chilliness of the back and abdomen, and who wore, besides two thick belts (maggördlar), not less than seven heavy shawls. A complete cure was obtained after she had gradually laid aside this excess of clothing, and had accustomed herself to sponging of the trunk with ice-cold water. A novel popular remedy is here introduced to our notice, viz. a bath in the freshlyplucked leaves of the birch tree, until perspiration breaks out over the whole body. Such popular remedies often succeed, when medical treatment has altogether failed. In the local palsies, so common in spinal irritation, Professor Huss has freely employed the endermic method, using strychnia in preference to any other remedy.

*Internal remedies.* Of antiphlogistics our author has only used mercury and tartar emetic, and these solely in plethoric subjects. The former was occasionally useful when pushed to salivation, but in many instances it utterly failed of effect. *Purgatives* he thinks absolutely necessary in most cases, to relieve the constipation and fæcal accumulation so common in females labouring under this disease, but those of the more drastic kind are decidedly to be rejected. The laxative mineral waters are, of all others, perhaps the most efficacious, though, for obvious reasons, they produce more benefit when the patient journeys to the springs. Much reliance is placed by Dr. Huss on *Tonics*, especially after fæcal accumulations have been removed, and of these he prefers the preparations of iron, while he considers quinine to have obtained an undeserved reputation in this disease. Many have regarded *narcotics* only as palliative remedies in spinal irritation. Our author is, however, deeply impressed with their great value as a means of cure. Small doses of opium or of morphia are decidedly the most successful means of treating the convulsive movements or tremors of the extremities; while cramp of a more tonic character, resembling tetanus, is equally amenable to this powerful drug. It should be given in small doses at first, and be continued for at least a fortnight to effect a cure. In cases of paralysis, strychnia has seemed to be a very valuable remedy, when given in doses of $\frac{1}{24}$th to $\frac{1}{12}$th of a grain two or three times a day. In one case marked good effects were obtained by the use of the Faba Sti. Ignatii. Belladonna proved only a palliative in cases of spasmodic asthma, from spinal irritation. From the preparations of arsenic, zinc, and copper, Dr. Huss has obtained no satisfactory results.

As an antispasmodic, the preparations of valerian deserve, he thinks, their reputation. The root, or rather *the fibrils of the root*, of Artemisia vulgaris, which appear to be the only active part of the plant, are much lauded by Dr. Huss for antispasmodic qualities. The root itself, he says, is nearly, if not quite, inert. Arnica is also in Sweden, as in Germany, a favorite remedy, especially in debilitated constitutions. Phosphorus and musk are favorably mentioned by our author, in convulsive affections, especially in laryngismus stridulus.

*Exercise and the due regulation of the diet* are strongly insisted upon by Dr. Huss; and he warns us against yielding to the unwillingness of patients to move, or to rise from their beds; as the completion of the cure can often only be obtained by exercise of the affected parts. From page 65 to 109 of the volume before us we are presented with a long and minute record of cases of spinal irritation; under which name our author seems to include more diseases than are generally ranked as such by English practitioners. Thus tetanus, chorea, and many forms of paralysis, are here described. One case of traumatic tetanus recovered under the free use of opium and of tartar emetic, one grain of each having been administered every hour. Of fifteen cases of tetanus (our author does not say they all were the consequence of wounds) five recovered under this plan of treatment.

Dr. Huss considers chorea to be a disease in which the spinal cord acts independently of the brain, and that the impulse in this malady is produced alike upon the extensor and upon the flexor muscles; while in tetanus the former only are affected. A kind of partial paralysis often, he says, continues long after severe attacks of either disorder. We would willingly extend to greater length our analysis of the interesting cases detailed here, but our notice of this malady will, perhaps, be already considered too long, we will, therefore, extract only one more observation relating to this portion of our subject:

"Rheumatism," says Dr. Huss, "is but too often made the scapegoat of many obscure complaints, and of paralysis among the rest. Partial paralysis may, however, occur from causes identical with those which produce rheumatism. Thus I have seen an individual who had placed his hand, at night, against a cold and damp wall, and on awaking in the morning that hand was completely paralysed. I have also treated a child which, for half an hour, had amused itself with melting ice on a frozen window with one of its fingers, and that member had become paralysed. In both cases I consider the first effect to have been produced on the cutaneous nerves, and to have been transmitted from thence to the spine; from whence, again, the paralysis was extended to the affected members."

In such cases we should, perhaps, have applied our remedies to the spine itself; but they both seem to have recovered by the endermic application of strychnia to the hands.

*Diseases of the respiratory organs.* Our author has already, in former reports, dwelt at great length upon these maladies; and his notice of them here is consequently short. Eight cases of diseases of the larynx and trachea were treated in the course of the year. One case occurred in a male patient, the other seven were females, and there were two deaths. The first was the cure of a girl, thirteen years of age, who had suffered from gradually increasing loss of voice and dyspnea for above a year; when suddenly, after exposure to a cold north-east wind, she was seized with acute laryngitis, which proved fatal, in spite of tracheotomy and all other remedies. On dissection, a mulberry-shaped excrescence was found occupying the ventriculus Morgagni of the left side, while the whole mucous surface of the trachea, and even down to the minute divisions of the bronchi, was clothed with a firm, false membrane, about half an inch in thickness. It is curious that the patient, some years before, had had a similar excrescence on the face, which entirely disappeared, according to the report of the child's parents, on the application of a popular Swedish remedy, which was once common, too, in England, viz. the hand of a corpse.

*Pneumonia.* The report of this year presents us with a brief summary of 140 cases, having a mortality of 10 per cent. Of these, 23 were females and 117 males, and by far the greater number were between 20 and 40 years of age. Inflammation of the lungs is surely more frequent in these northern regions than in Great Britain. The months of April and May have for many years afforded in Sweden more than one third of the cases of pneumonia, and the mortality was highest in June and July, which Dr. Huss ascribes to the great frequency of various abdominal complications in the early part of the summer. The relative seat of the inflammation was as follows:

Pneumonia of the right lung in 87 cases
,, left lung in 39 cases
,, both lungs in 14 cases.

A great disproportion was observed in the mortality of the two sexes, the deaths among the males averaging 1 in 14, and among the females 1 in 4. Pneumonia in the left side is considered by Dr. Huss as much less fatal than pneumonia of the right, the proportions being 1 death in 19 in the former case, and 1 in 10 of the latter form. Dr. Huss acknowledges that he can assign no reason for this difference. His general experience has taught him that females are more liable to double pneumonia, which is always the most fatal.

The treatment of *tubercular phthisis* appears to be in Sweden, as elsewhere, an ever disputed point. Dr. Huss fancies that he has lately obtained more benefit from moxas applied below the clavicles, than from any other mode of counter-irritation. For his general opinions regarding this malady we are referred to the Report of the preceding year.

*Diseases of the circulating system.* In the space of two years, 28 cases of acute pericarditis have occurred in the hospital, and of these not less than 23 were females. Unfortunately Dr. Huss again refers us to the Reports of preceding years for the details of his cases, and the mode of treatment employed. Under the head of phlebitis there is given here a well-observed instance of slow destruction of life by this malady after childbirth. Acute symptoms did not appear until the fourth week, and the patient lingered till the thirteenth week after delivery.

Dr. Huss allows of at least two causes of phlegmasia alba dolens: the one is the compression of the hypogastric and common iliac veins by the head of the infant, the other arises from pus circulating in the system. The former usually comes on soon after delivery; the latter often does not appear till the second or third week.

*Diseases of the digestive organs.* Simple ulcerations of the stomach are treated by our author by rest, light farinaceous diet, and the salts of silver, copper, or zinc, while counter-irritation is applied externally. He confesses, however, that the diagnosis of this malady during life is extremely difficult, and the great obscurity of the symptoms will cast a considerable shade of doubt over every recorded case of cure.

*Typhoid fever.* Dr. Huss considers the name of *enteritis folliculosa* as by no means universally applicable to the disease in question. He adds his testimony to that of almost all medical writers in this country, that typhus fever is most rife and severe where noxious exhalations from sewers, cesspools, &c., especially abound. During wet winters it has been remarked in Sweden that typhus was very common; but as soon as the long and steady frosts of these northern regions commenced, there ensued a great and striking abatement of the disease. We fear that travellers have been deceived by the beautiful islets and clear waters of the Swedish capital. "There is no capital in the world, at least, we think, in Europe, where so much refuse is allowed to lie in the open streets, and, above all, in the large open courts of the dwelling-houses (gårdar)."

The number of typhus-fever patients in 1842 was nearly double that of

1841, in consequence of an epidemic having broken out at an early period of the year, and which supplied patients to the hospital at the rate of 50 cases per month. The total number of cases was 517, of which 342 were males and 175 females. The mortality was 10 per cent., i. e. 1 in 12 of the males, and 1 in 8 of the females, and, as usual, it was highest in those of advanced age. Of the whole 517, a preponderance of cerebral symptoms was observed in 136 cases; in 208, abdominal symptoms prevailed; and in 173 patients the signs of both affections were combined. The mortality was much the highest among those who exhibited prominent cerebral symptoms, and lowest where the two affections appeared together. Death occurred, too, at a much earlier period in cerebral cases.

The following is an abstract of an analysis of various symptoms of typhoid fever:

*a.* Dr. Huss insists much upon the withered loose condition of the heart in all cases of death from typhus, the muscular substance of that organ having entirely lost its firmness and elasticity. He suggests that to this loss of power of the heart is to be attributed the pulmonary congestion so constantly observed in typhoid fever.

*b.* Disintegration of the blood was observed in 47 cases out of 54 that were examined with this view. Dr. Huss remarks that, when death occurs after the third week, the blood is often found to have regained a certain portion of its fibrine, perhaps in consequence of the numerous secondary inflammations that are then liable to occur.

*c.* The spleen also, which, in typhoid fever, is generally found softened and enlarged, changes, in protracted cases, so as to regain its natural size, though its texture becomes more firm and granular than in the normal state. A softened and enlarged spleen was most frequent in those who died with predominant symptoms of cerebral disturbance.

*d.* The peculiar typhoid ulcerations of the intestinal canal were found in 39 cases out of 54, which is certainly a larger proportion than generally occurs in England. In 1841 the proportion in Stockholm was as 6 to 1; this year it was 3½ to 1. Dr. Huss adheres still to the opinion he expressed in former Reports, that in proportion as the typhoid exanthema shows itself more on the external surface of the body, so is it diminished in the intestines.

At page 151 we find an interesting account of the epidemic typhus fever which prevailed in December, 1841, and January, 1842, in the police barracks (gendarmerie) at Stockholm. Of 280 men constituting this portion of the force, 64 were affected with typhus fever in the space of six weeks. Thirteen cases illustrating the three classes of typhoid fever before mentioned are here given at great length, and they are followed by an extended analysis of the symptoms of the disease. The cases of most frequent occurrence in this epidemic were of the mixed class, or those wherein the cerebral and abdominal symptoms were of equal intensity, and Dr. Huss here repeats his firm conviction, that all the three classes or varieties of typhoid fever here named are alike the results of a similar morbid process, and are therefore to be considered as one and the same disease.

*a.* Cerebral form (page 167). Dr. Huss divides his history of the symptoms into two periods. In the first, along with the usual indications of cerebral typhoid fever, hemorrhage from the nose frequently occurred, but it does not seem to have exercised any peculiar influence on the duration or character of the malady.

A species of typhoid eruption was observed in all the cases, appearing as light red streaks (not the *tâches rosées lenticulaires*) over the chest and shoulders, and spreading from thence over the whole body, but rarely extending to the face. These streaks exhibited a tendency to assume a deeper hue, and at length, in the severest cases, they became of a deep violet, and the intensity of their colour was in exact proportion to the severity of the symptoms.

*b*. Abdominal typhus. In this form the usual well-known symptoms appeared, except that we do not find any distinct mention of the roseate lenticular spots so common in this type of fever. The typhoid exanthema, (?) however, before spoken of, was here again observed, but it never assumed the deep blue or violet colour. In four cases there were, however, spots like those of purpura, which *did not* disappear on pressure. When much diarrhea had occurred, there was generally excessive emaciation, and the recovery was exceedingly slow.

Dr. Huss was often able to foretell the approach of convalescence from the fact of the patient's assuming a posture different from that which they had kept throughout the severity of the disease, which had been generally the recumbent position on the back.

After a minute historical analysis of these three types of typhoid fever, Dr. Huss proceeds to consider the various peculiarities of this epidemic.

1. *Varieties.* Under the first head he acknowledges that there was often considerable difficulty in determining the exact species of fever, as at one time abdominal, and at another cerebral symptoms predominated in the same case. Sometimes, too, abortive cases occurred, where the fever came on severely at first, but disappeared suddenly upon an emetic or purgative being given, and without running its usual course. All epidemics present numerous instances of this kind, but it is still uncertain whether these cases would have become really severe if they had been left entirely to nature.

2. *Complications* occurred but rarely, and were chiefly confined to affections of the respiratory organs. It is remarkable that those of the gendarmerie who had previously exhibited symptoms of phthisis were peculiarly free from the influence of the epidemic.

3. *Sequelæ.* In two cases where the cerebral symptoms had greatly predominated, almost total deafness ensued, and continued for a long period. Œdema of the legs and feet was very frequent where the heart's action was so much weakened, that the first sound was scarcely audible, but it yielded rapidly to tonic treatment.

4. *Terminations and prognosis.* Critical symptoms, especially prolonged sleep and copious perspiration, occurred in twenty-nine cases, or in nearly one half of those affected, and Dr. Huss considers the tendency to crises to have been here much greater than in ordinary typhoid fever.

5. *Causes.* Two thirds of the cases occurred in individuals under 30 years of age. We have, in this particular instance, peculiar advantages for ascertaining the influence of age, temperament, &c., in exciting disease, as all the individuals affected were submitted to the same conditions of life, and all, too, were equally exposed to a similar routine of duty.

One principal exciting cause of the disease is thought to have been the constant rain during the night, when the guard was patrolling, and this severe weather lasted almost without intermission for above two months. The men came in from patrol (a duty of ten to eleven hours' duration) with their clothes perfectly saturated with rain, and their moist garments were hung up to dry in the crowded room in which they slept, which was moreover raised to a high temperature to facilitate the process of desiccation. By this heat and moisture, with the exhalations from so many human beings congregated in one apartment, our author thinks that a typhoid miasm was at length generated. An immediate mitigation of the violence of the symptoms was the consequence of a change of situation, effected by removing the patients to a purer air and to better ventilated apartments, while the cases that subsequently appeared were all of a milder character. The disease did not appear to be of a contagious nature, as it did not spread in a single instance among the other patients, when the sick men were removed to the general hospital. Those who had served in the patrol for only a few weeks or months were as susceptible of the disease as the veterans, who had been for years in the force.

6. *Nature of the malady.* Dr. Huss believes this epidemic to have consisted in an alteration of the blood, both in its physical constitution and in its vital properties, occasioned by the influence of the miasm brought into contact with it in the capillaries of the lungs. A similar epidemic is stated to have occurred among the workmen employed in excavating a canal in the Royal Park at Stockholm, during the months of August and September. In this instance the works were for some time carried on in a deep morass. It is to be regretted that no chemical investigation of the blood of the individuals affected by this epidemic was attempted, for, although few results of great immediate importance have hitherto been obtained by these researches, still it is obviously the line by which great discoveries are to be made, and every additional observation will prove of value. The quantity of serum was apparently in most cases diminished, the coagulum was loose and imperfect, and sometimes did not form at all, indicating a great deficiency of fibrine. In the two fatal cases the usual symptoms of typhoid action were observed—the fluid condition of the blood, the softened spleen, the atrophied state of the heart, and the swollen appearance of the glands of Peyer and Brünner in the abdomen; from which, however, as Dr. Huss observes, we are by no means to infer that these glands were so affected in all those cases which recovered. The more robust the patient, the more intense in general were the cerebral symptoms, while the abdominal type predominated in those of a lymphatic and less sanguine temperament.

7. *Treatment. External remedies.* Venesection was only practised at the commencement of the malady, when there was much evidence of congestion in the brain and lungs. Beyond the fourth day it was never resorted to. When the abdomen was painful on pressure, especially over the iliac regions, cupping over that part was frequently practised with success, and was occasionally repeated up to the seventh day; but not after that period. Blisters, sinapisms, and turpentine epithems, particularly the last, were found to be of great benefit as derivatives, and, above all, were useful in counteracting the frequent tendency to congestion in the lungs and trachea. Ice, in many cases, was agreeable to the patient, as an external application; but wherever it irritated rather than soothed the sick, it was wisely discontinued.

*Internal remedies.* Purgatives (not of the drastic kind) were freely given in all forms, unless the diarrhea and abdominal complication was excessive; but Dr. Huss derived still greater advantages from the free use of the mineral acids, especially of the muriatic acid in decoct. altheæ, and this he continued to give as long as the pulse remained full (whether its beats were sharp or soft), and the sounds of the heart normal. The varied state of the tongue, or an increase of tenderness in the bowels, formed no contraindication to its use; but it could not be employed where a tendency was exhibited to congestion of the lungs or bronchi. When the pulse became small, and the first sound of the heart weak and short, so as nearly to resemble the second sound of that organ, the mineral acids were no longer available; and Dr. Huss had then recourse to phosphoric acid, which he combined with infusion of ipecacuan, where diarrhea indicated the presence of abdominal ulceration. Camphor was found to be very valuable in all those cases where the shortening of the first sound of the heart, and the diminution of its intensity, indicated debility of that organ. Dr. Huss considers this symptom, when it occurs, to be a sure indication for the employment of camphor, *and the only real one;* for no certainty can, he thinks, be obtained in this respect from the appearance of the tongue, from the pulse, or from the nature of the delirium. Where, however, there was diarrhea, accompanied with a deep flesh-red tongue, and also tenderness on pressure of the abdomen, he observed that the use of this drug often gave rise to vomiting and colicky pains in the abdomen. Might not the combination with hyoscyamus have obviated this inconvenience?

Musk is thought by our author to be indicated when the patient lies motionless on his back, with low, muttering delirium, plucking of the bedclothes, and subsultus tendinum; when the pulse is small and scarcely to be felt, and, above all, when the first sound of the heart is no longer to be distinguished. The doses must be large (three to five grain several times a day), or no good results can be expected.

Our author speaks favorably of opium, when the delirium is of a more violent character; but the skin must be cool and soft, and there must be no other signs of cerebral congestion. Belladonna may be employed in similar cases; but it seldom produces any effect until its well-known influence on the pupils is developed. Sulphuric acid was employed with favorable results during convalescence, especially when the vital powers were much impaired; as also in the diarrhea which sometimes persisted long after the febrile symptoms had disappeared.

Dr. Huss considers the employment of the ethereal oil of turpentine to be one of the greatest improvements in the treatment of certain complications of typhoid fever. It is of essential value, he says, in the typhoid form of pneumonia, which is so apt to show itself between the eleventh and fourteenth day of the disease; particularly in those cases where the debility of the heart's action is announced by the absence or diminution of the first sound of that organ. The weakened heart is then unable to propel the blood through the capillaries of the lungs, congestion ensues, and proceeds to inflammation of a low typhoid kind. The other indications for the employment of turpentine, in this complication, are cough with viscid and more or less bloody expectoration, dulness on percussion over some portion of the chest, particularly at the back part, with crepitation and tubar, or bronchial respiration there. Dr. Huss gives the following formula as a convenient mode of administering this medicine: ℞. Ætherol. terebinth. ℥ss; vitell. ovor. No. j; mucilag. gumm. Arab. ℥iij; Aq. destillat. ℥j; M. ft. emulsio. signr. One teaspoonful to be given every hour in the daytime, and one grain of opium, in two doses, during the night.

8. *Duration.* The average of the whole cases gives twenty-three days as the mean duration of each case.

We regret that we cannot devote more space to the remaining contents of this volume, which chiefly relate to various diseases of the digestive organs. A curious instance is given (along with a well-executed engraving) of adipose tumours occupying a rather unusual seat, viz. the inner surface of the small intestine. These tumours, about twelve in number, hung by a small pedicle from the internal surface of the ileum. They were composed of adipose cells, inclosed in a common cyst, and covered by the mucous lining of the intestine. Their origin, therefore, was probably in the submucous cellular tissue.

The Report of this year is very brief in regard to diseases of the liver. An interesting history is detailed at great length of a man who, during life, had suffered from that rare form of hemorrhoidal tumour, the erectile (described by Earle and Copeland), and in whom, after death, the liver was found studded with tumours of a similar character. Dr. Huss considers these latter to be fully entitled to the much-disputed appellation of hemorroids of the liver ("hæmorrhoider på lefvern"); but it seems doubtful whether the original tumours in the rectum can fairly claim that title.

Our author is, we think, somewhat imbued with the doctrines of Schönlein regarding the influence of the portal system in the production of disease. In one case, which he relates, there was found cirrhosis of the liver to a high degree, while the vena porta was much dilated; and on its inner surface there were observed numerous bony cicatrices, similar to those so often seen in the dilated aorta, while the veins opening into the vena porta presented the like appearance. These alterations Dr. Huss does not allow to be the result of primitive inflammation in these blood-vessels; but he maintains that the cirrhosis of the liver was the primary disease, which, by obstructing the free flow of blood through the vena porta, caused dilatation of that blood-vessel; and this, when long continued, produced a chronic inflammation, with thickening and deposition of osseous matter.

We trust that this brief analysis of Dr. Huss's Report will not have been uninteresting to our readers, and that, in common with ourselves, they will wish for some further acquaintance with this author's writings. A patient spirit of investigation characterizes the whole volume; and while Dr. Huss, in this respect, may take his stand with the most diligent pathologists of Germany, he is far superior to the brilliant writers of France in his intimate acquaintance with the medical literature of other countries.

